# Supplementary material for: Salivary proteins potentially derived from horizontal gene transfer are critical for salivary sheath formation and other feeding processes
Source: Commun Biol. 2024 Mar 2;7:257. doi: 10.1038/s42003-024-05961-9 (PMC10908841; doi:10.1038/s42003-024-05961-9)
Supplement: Supplementary file 2 — Supplementary Material [file 42003_2024_5961_MOESM2_ESM.pdf]

Supplementary Information for

Salivary proteins that potentially derived from horizontal gene transfer are critical for salivary sheath formation and other feeding process

Hai-Jian Huang [#, *, 1], Li-Li Li [#, 1], Zhuang-Xin Ye [1], Jia-Bao Lu [1], Yi-Han Lou [2], Zhong-Yan Wei [1], Zong-Tao Sun [1], Jian-Ping Chen [1], Jun-Min Li [1], Chuan-Xi Zhang [*, 1]

[1] State Key Laboratory for Managing Biotic and Chemical Threats to the Quality and Safety of Agro-Products, Key Laboratory of Biotechnology in Plant Protection of Ministry of Agriculture and Zhejiang Province, Institute of Plant Virology, Ningbo University, Ningbo 315211, China

[2] Zhejiang Provincial Center for Disease Control and Prevention, Hangzhou 310051, China

[#] These authors contribute equally to this work

[*] Corresponding authors: Hai-Jian Huang, huanghaijian@nbu.edu.cn; Chuan-Xi Zhang, chxzhang@zju.edu.cn

Supplementary Figures: 1-8

Supplementary Tables: 1-7

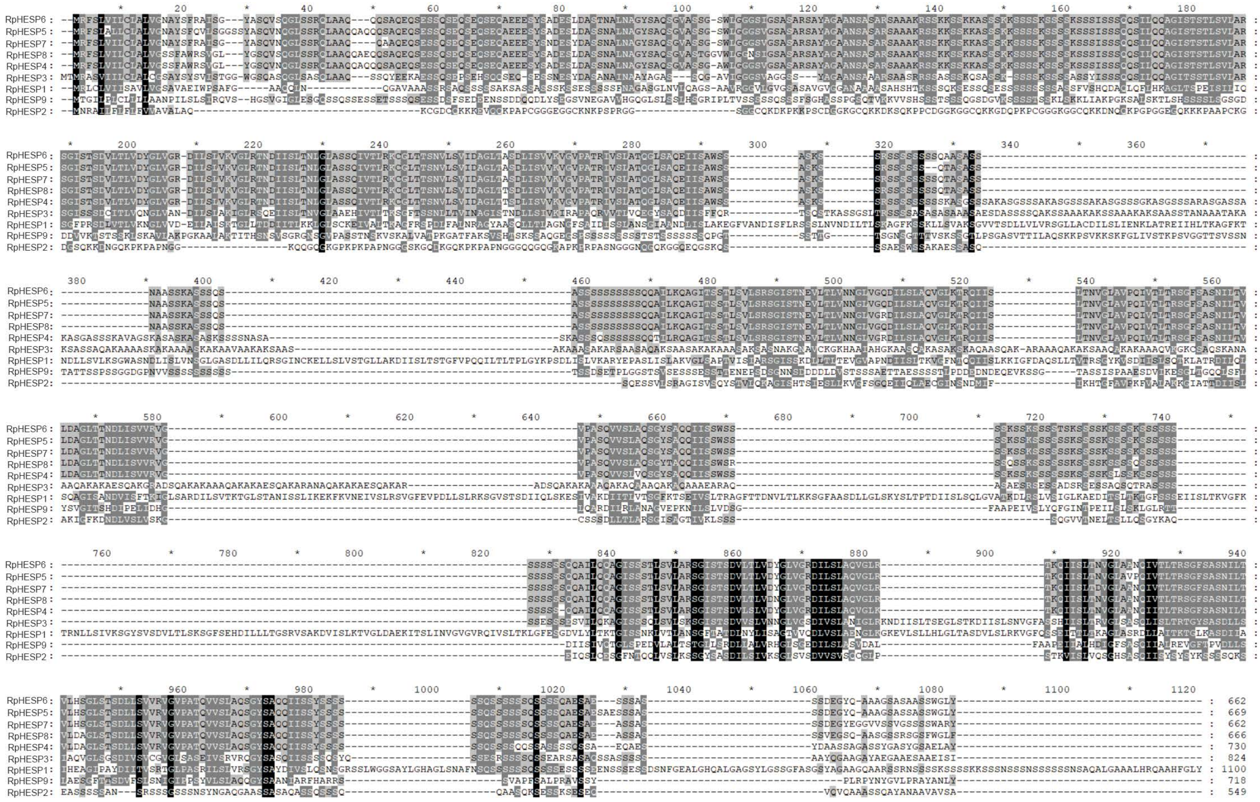

Supplementary Figure 1. Amino acid alignments of RpHESPs. Amino acid sequences of nine RpHESPs were aligned using ClustalX software. Black shades indicate the conserved regions of RpHESPs.

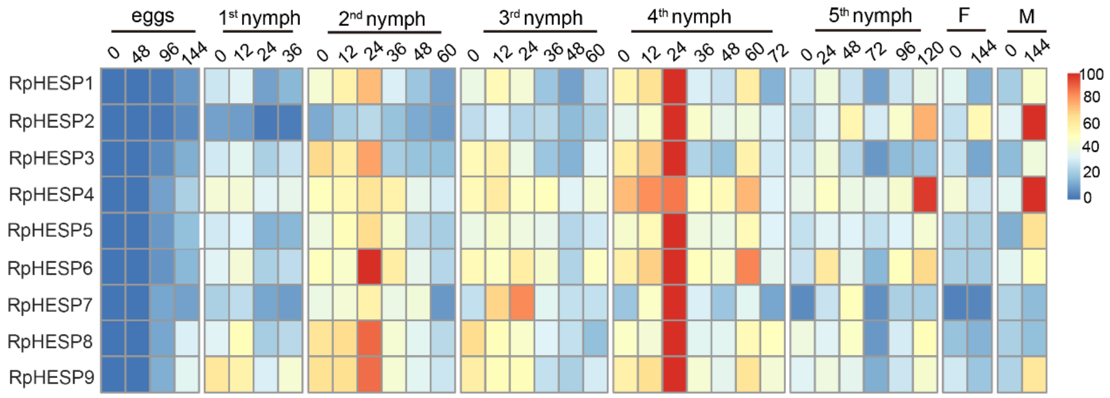

Supplementary Figure 2. Expression patterns of RpHESPs in different development stages. The expression patterns of nine RpHESPs in 37 developmental stages were calculated based on transcriptomic data and illustrated by a heat map.

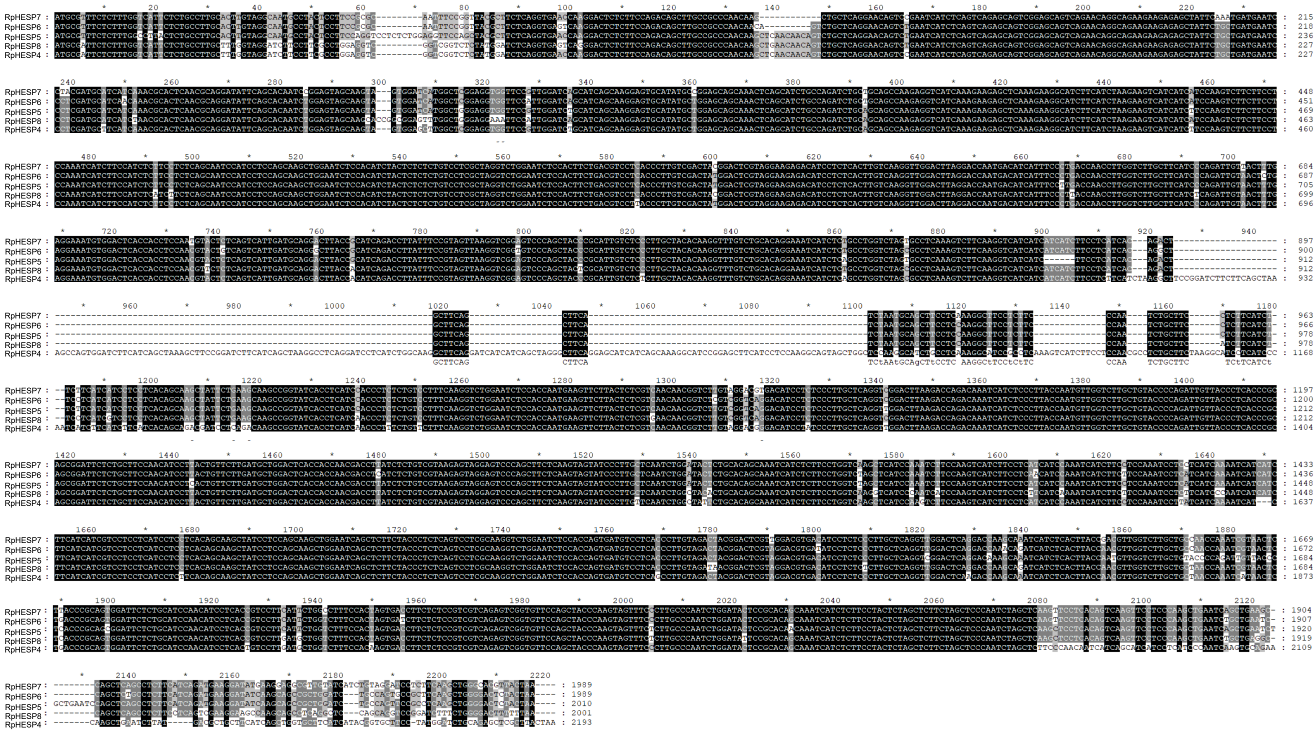

Supplementary Figure 3. Nuclear acid alignments of RpHESP4 to RpHESP8. Nuclear acid sequences of RpHESP4 to RpHESP8 were aligned using ClustalX software. Black shades indicate the conserved regions.

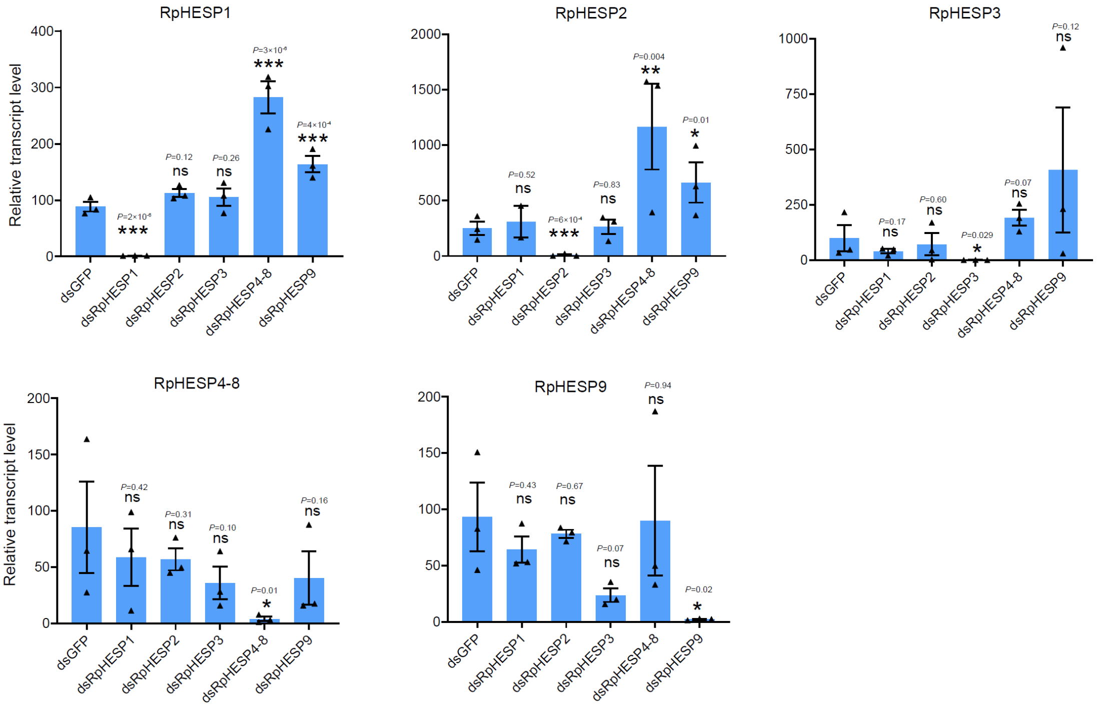

Supplementary Figure 4. Silencing efficiency and specificity of RNA interference in *Riptortus pedestris*. ds*RpHESP1*, ds*RpHESP2*, ds*RpHESP3*, ds*RpHESP4-8*, and ds*RpHESP9* were injected into the third instar nymphs, respectively. At four days post-infection, Relative transcript levels of *RpHESPs* were determined by qRT-PCR. Data are presented as mean ± SEM (n=3). *P*-values between ds*RpHESP* treatments and ds*GFP*-treated control were determined by two-tailed unpaired Student's *t*-test. ***$P < 0.001$; **$P < 0.01$; *$P < 0.05$; ns, not significant.

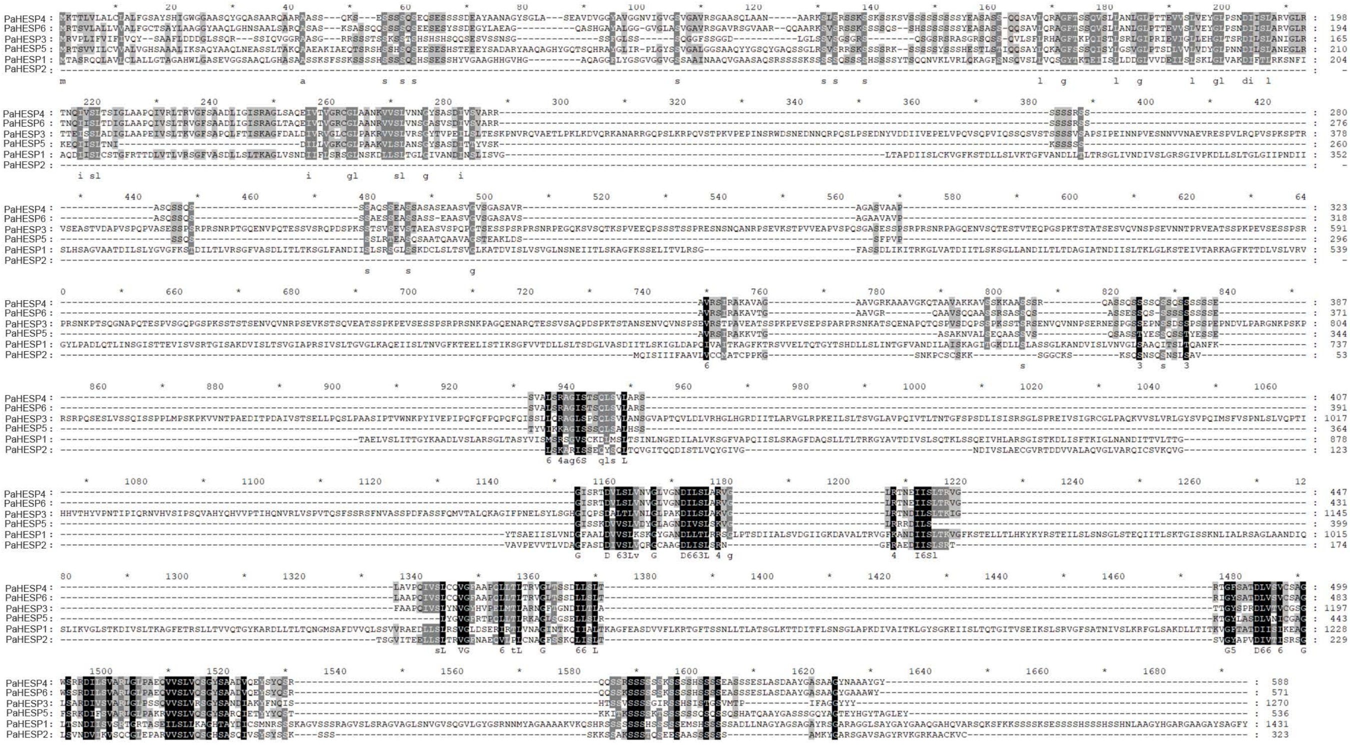

Supplementary Figure 5. Amino acid alignments of PaHESPs. Amino acid sequences of 6 PaHESPs were aligned using ClustalX software. Black shades indicate the conserved regions of PaHESPs.

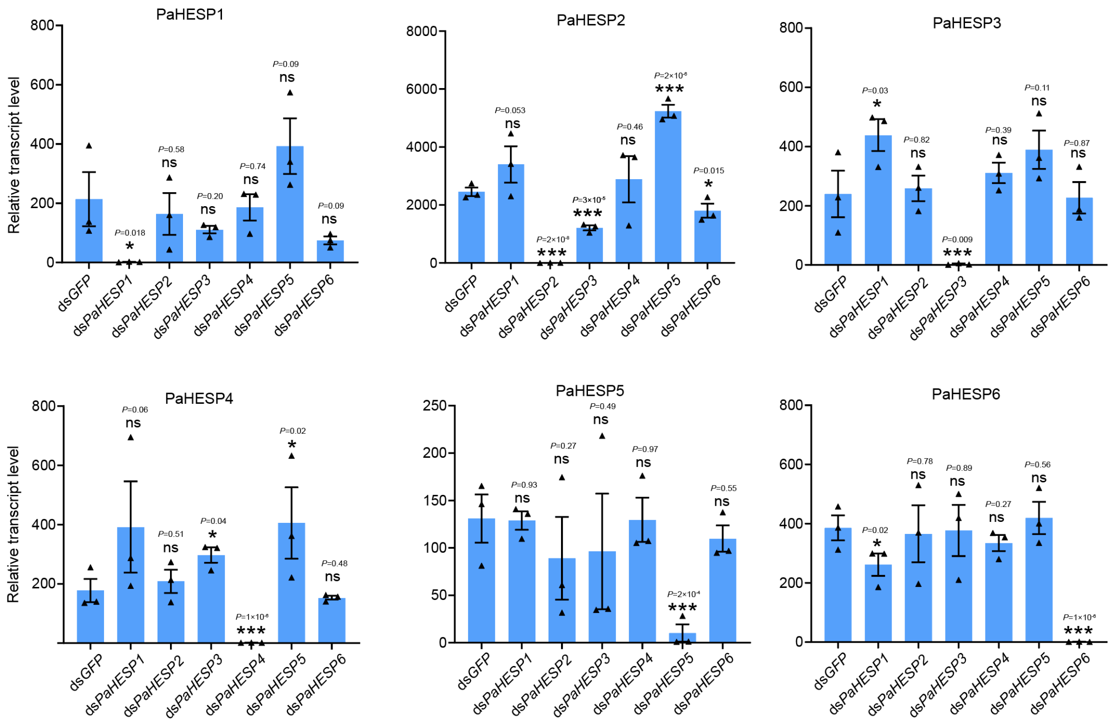

Supplementary Figure 6. Silencing efficiency and specificity of RNA interference in *Pyrrhocoris apterus*. ds*PaHESP1*, ds*PaHESP2*, ds*PaHESP3*, ds*PaHESP4*, ds*PaHESP*5, and ds*PaHESP*6 were injected into the third instar nymphs, respectively. RNAi efficiency was determined at four days post-injection using qRT-PCR. Data are presented as mean ± SEM (n=3). *P*-values between ds*RpHESP* treatments and ds*GFP*-treated control were determined by two-tailed unpaired Student's *t*-test. ****P* < 0.001; ***P* < 0.01; **P* < 0.05; ns, not significant.

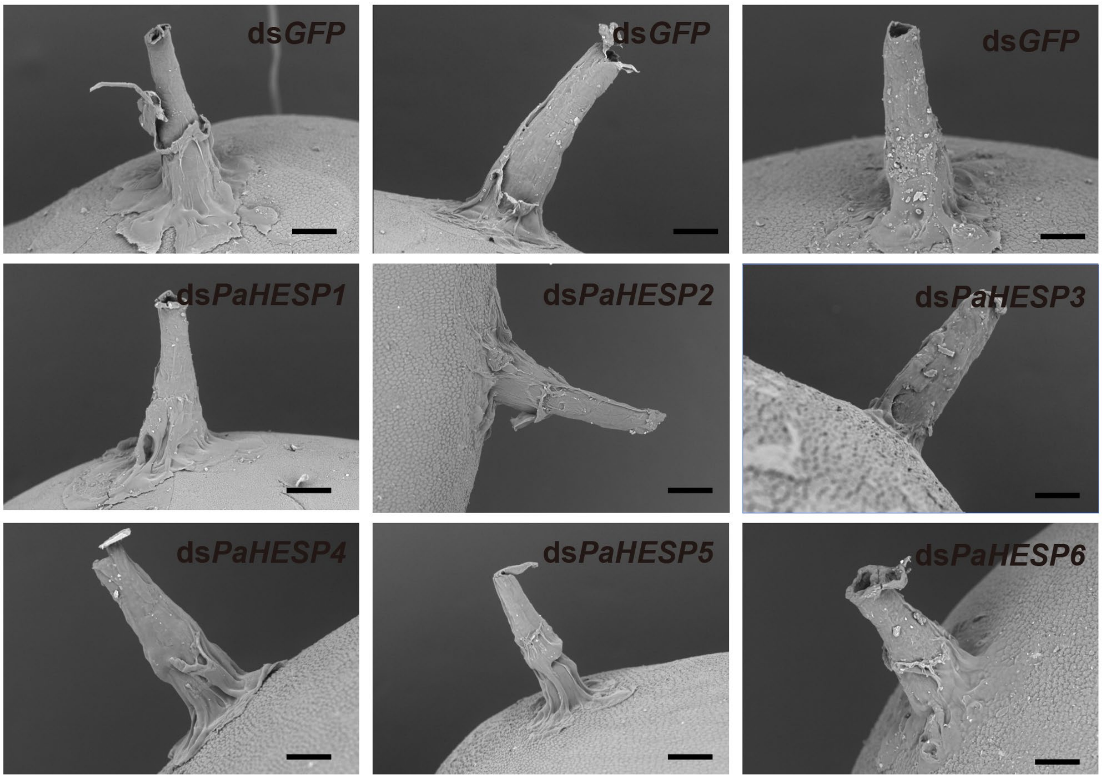

Supplementary Figure 7. Effects of ds*PaHESP* treatments on salivary sheath formation in *Pyrrhocoris apterus*. The morphological changes of salivary sheath secreted by ds*PaHESP*- were observed under scanning electron microscopy. No significant change in salivary sheath morphology was detected under ds*PaHESP* treatments compared with ds*GFP*-treated control. More than 20 salivary sheaths were tested, and similar results were found. Bar = 40 μm.

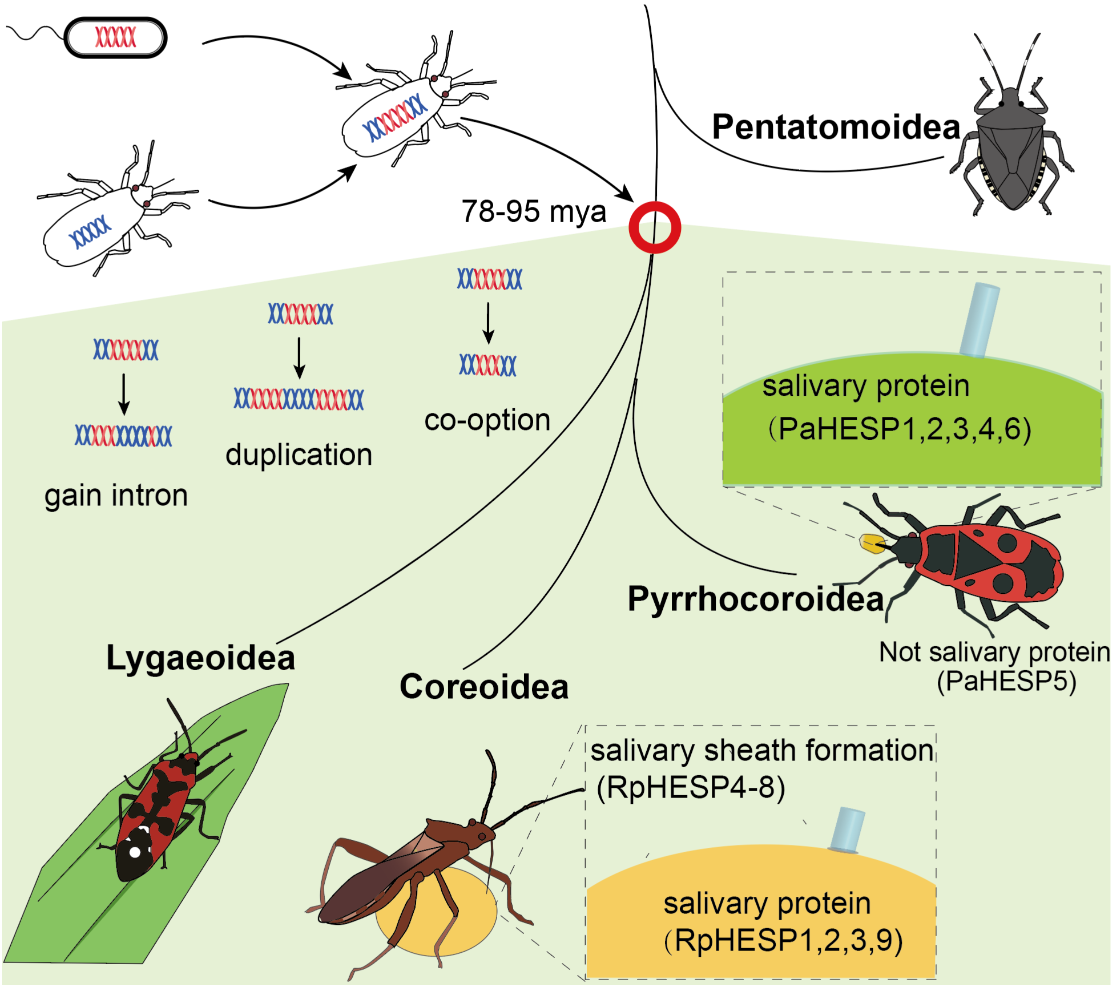

Supplementary Figure 8. The proposed model for HESP evolution. The HESP was potentially transferredfrom bacteria to an ancestral bug of Coreoidea, Lygaeoidea, and Pyrrhocoroidea superfamily. The acquired genes underwent duplications, gained introns, and evolved through co-option. In the case of *Riptortus pedestris,* all of the RpHESPs were salivary proteins that were secreted in large amounts into plants. The RpHESP4 to RpHESP8 proteins were critical for insect survival as they were involved in the formation of the salivary sheath. The other RpHESPs, however, promote the performance of *R. pedestris* in other unknown manners. In *Pyrrhocoris apterus,* six PaHESP were originated from a single HGT event. Among them, five PaHESPs were secreted into plants during insect feeding, while the PaHESP5 no longer functioned as a salivary protein after long-term evolution.

Supplementary Table 1 Peptides of RpHESPs identified in infested soybean seed using LC-MS/MS

Gene	Unique peptide		Peptides[2]
	Number[1]	Coverage	
RpHESP1	63	62.3%	AEDITSLTK; AGFTTDNVLTLK; AGFTTDNVLTLKK; AGLTSPEISILIQSGFPR; ARYEPASLISLAK; ASDIIAIHEAGIPAYDIITVSR; DIISLTSTGFVPQQILTLTPLGYKPSDLISLVK; DIITLVTSGFK; DILQLSQAGISANDVISFTK; DILSVTK; DLLAITK; DLLTLTEVGVAPNDIISLTK; EGFVANDISFLR; EIIHLTK; EIVALTK; ELLSLVSTGLLAK; EVLSLLHLGLTASDVLSLR; FKVNEIVSLR; GKEVLSLLHLGLTASDVLSLR; IGFDAQSLLTVTR; ITSLINVGVGVR; KIGFDAQSLLTVTR; KSGFAASDLLGLSK; KSGVSTSDIIQLSK; KSSSNSSSNSSSSSSNSAQALGAAALHR; KVGFQSSEITTLTK; LGFESGDVLYLTK; NLLSIVK; PSDLISLVK; QAAHFGLY; QIVSLTK; SAQSSSSSAKSASSASSSKSSESSSSSFNAGASGLNVLQAGSAAVR; SDLVTLVK; SESSQSESSSSSSSSSASSFVSHQDAQLQFLHK; SGFAASDLLGLSK; SGFSEHDILLLTGSR; SGVSTSDIIQLSK; SGVVTSDLLVLVR; SGYSAYDIVSLQSNSGR; SGYSVSDVLTLSK; SLVSIGLK; SPDLFALNR; SSESSSSSFNAGASGLNVLQAGSAAVR; SSSLNVNDILTLSK; SSSNSSSNSSSSSSNSAQALGAAALHR; SVGFEVPDLLSLR; SVGFEVPDLLSLRK; TGFSSSEIISLTK; TGLLTDDIITLK; TGLLTDDIITLKK; TGLPASR; TGLSTANISSLIK; TNDLLSVLK; TSEIVSLTR; TVGLDAEK; VGFNTQQIISLK; VGFNTQQIISLKK; VGFQSSEITTLTK; VGLSAPTVISIAR; VNEIVSLR; VSDIISLSQTK; YEPASLISLAK; YSLTPTDIISLSQLGVATK
RpHESP2	14	34.4%	AESSASQSQESSVLSR; AGISHTSIESLLK; AGISVSQYSTVLQK; EVQQKPAPCGGGEGGCK; GCSSSDLLTLAR; GIATTDIISLAK; HTGFAVPK; IGFKDNDLVSLVSK; KGIATTDIISLAK; LSSSSQGVVTNELTSLLQSGYK; PAPNGGGSKGQDKGQK; QSSSAESWSSAK; SGISAGTIVK; VISLVQSGHSASQIISYSYSYK
RpHESP3	11	23.4%	AGISSSQLSVLSK; AQAAAQAKAQAAAEAR; ASAKSKAQAASQAK; ASSSSSSESSSESVILQK; KNDIISLTSEGLSTK; QGYSASQIISSSSQSYQSSESR; SAASSTANAAATAK; SQEIISLTNVGLAAEHIVTLTK; SSSSASASASAAASAESDASSSSQAK; VGLSASQLISLTR; VVTLVQEGYSAQDIISFFQR
RpHESP4	21(15)	54.4%	ASGSSSAKASGSSSAKASGSSSGK; ASSSQSSSSSSQQTILR; QIISLTNVGLAANQIITLTR; SGISTSDVLSLVDYGLVGR; SVGLYGSQVNQGLSSR; VGVPASQVVSLVQSGYSAQQIISSWSSSSK; SAYAGAANSASAR; SSSISSSQQSILQQAGISTSTLSVLAR; SGISTSDVLTLVDYGLVGR; DILSLVK; TNDIISLTNLGLASSQIVTLR; TNDIISLTNLGLASSQIVTLRK; VGVPATR; IVSLATQGLSAQEIISAWSSASK; QAGITSSTLSVLSR; QIISLTNVGLAVPQIVTLTR; SGFSASNILTVLDAGLTTNDLISVVR; SGISTNEVLTLVNNGLVGR; DILSLAQVGLK; CGLTTSNVLSVIDAGLTTSDLISVVK; SGFSASNILTVLDAGLSTSDLLSVVR
RpHESP5	19(17)	57.4%	SSSSSSQTASASSNAASSK; TKQIISLTNVGLAVPQIVTLTR; ASSSQSASSSSSSSSSSQQAILK; DILSLAQVGLR; SAYAGAANSASAR; SSSISSSQQSILQQAGISTSTLSVLAR; SGISTSDVLTLVDYGLVGR; DILSLVK; TNDIISLTNLGLASSQIVTLR; TNDIISLTNLGLASSQIVTLRK; VGVPATR; IVSLATQGLSAQEIISAWSSASK; QAGITSSTLSVLSR; QIISLTNVGLAVPQIVTLTR; SGFSASNILTVLDAGLTTNDLISVVR; KCGLTTSNVLSVIDAGLTASDLISVVK; VGVPASQVVSLAQSGYSAQQIISSWSSSSK; SGFSASNILTVLHSGLSTSDLLSVVR; SGISTNEVLTLVNNGLVGQDILSLAQVGLK

RpHESP6	20(18)	60.4%	AISGYASQVSQGLSSR；SSSSSSSSQAASASSNAASSK；ASSSQSASSSSSSSSSSQQAILK; DILSLAQVGLR; SAYAGAANSASAR; SSSISSSQQSILQQAGISTSTLSVLAR; SGISTSDVLTLVDYGLVGR; DILSLVK; TNDIISLTNLGLASSQIVTLR; TNDIISLTNLGLASSQIVTLRK; VGVPATR; IVSLATQGLSAQEIISAWSSASK; QAGITSSTLSVLSR; QIISLTNVGLAVPQIVTLTR; SGFSASNILTVLDAGLTTNDLISVVR; KCGLTTSNVLSVIDAGLTASDLISVVK; VGVPASQVVSLAQSGYSAQQIISSWSSSSK; SGFSASNILTVLHSGLSTSDLLSVVR; QIISLTNVGLAANQIVTLTR; SGISTNEVLTLVNNGLVGQDILSLAQVGLK
RpHESP7	20(19)	57.4%	AISGYASQVNQGLSSR; SSSSSSSSQTASASSNAASSK; ASSSQSASSSSSSSSSSQQAILK; DILSLAQVGLR; SAYAGAANSASAR; SSSISSSQQSILQQAGISTSTLSVLAR; SGISTSDVLTLVDYGLVGR; DILSLVK; TNDIISLTNLGLASSQIVTLR; TNDIISLTNLGLASSQIVTLRK; VGVPATR; IVSLATQGLSAQEIISAWSSASK; QAGITSSTLSVLSR; QIISLTNVGLAVPQIVTLTR; SGFSASNILTVLDAGLTTNDLISVVR; SGISTNEVLTLVNNGLVGR; DILSLAQVGLK; KCGLTTSNVLSVIDAGLTASDLISVVK; VGVPASQVVSLAQSGYSAQQIISSWSSSSK; SGFSASNILTVLHSGLSTSDLLSVVR
RpHESP8	20(18)	55.6%	SGISTNEVLTLVNNGLVGQDILSLAQVGLK; DILSLAQVGLR; SAYAGAANSASAR; SSSISSSQQSILQQAGISTSTLSVLAR; SGISTSDVLTLVDYGLVGR; DILSLVK; TNDIISLTNLGLASSQIVTLR; TNDIISLTNLGLASSQIVTLRK; VGVPATR; IVSLATQGLSAQEIISAWSSASK; QAGITSSTLSVLSR; QIISLTNVGLAVPQIVTLTR; SGFSASNILTVLDAGLTTNDLISVVR
RpHESP9	11	19.6%	ALVATPK; AVSSYPLRPYNYGVLPR; DLLALVR; GNSGVPASSTNSK; IPLTVSSSSSQSSSFSGHASSPGSQTVK; KVVSHSSSTSSSQGSDGVK; LANAGVEPK; SSVAPFSALPR; TITHSNSVSGR; TLSHSSSSLSGSGDDDVVK; VVSHSSSTSSSQGSDGVK; SVGLYGSQVSQGLSSR; SGISTSDVLTLVDNGLVGR; CGLTTSNVLSVIDAGLTTSDLISVVK; SSSSSSSSQTASASSNAASSK; SGFSASNILTVLDAGLSTSDLLSVVR; QIISLTNVGLAANQIVTLTR; ASSSQSASSSSSSSSSSQQAILK;

[1] The number in the brackets indicates unique peptides belonging to more than one protein.

2 Unique peptides belonging to more than one protein are labelled in red.

Supplementary Table 2 Genomic location of HESPs and their adjacent genes

	Gene ID	Gene loci[1]	Description	Best matched species in NCBI [2]	E-value	Classification
Riptortus	chr1.0265	Chr1:8894502-8895218	piggyBac transposable element-derived protein	*Pararge aegeria*	3e-43	Insects
pedestris	chr1.0266	Chr1:8931822-8940069	cathepsin B	*Oryctes borbonicus*	8e-25	Insects
**Region1**	chr1.0267	Chr1:8944559-8955924	cathepsin B	*Halyomorpha halys*	1e-89	Insect
	chr1.0268	Chr1:8958856-8960890	box C/D snoRNA protein	*Biomphalaria glabrata*	3e-105	Snail
	chr1.0269	Chr1:8968857-8975311	*Riptortus pedestris*-specific protein	-	-	-
	chr1.0270	Chr1:8988221-8998552	RpHESP9	*Deltaproteobacteria bacterium*	3e-17	Bacteria
	chr1.0271	Chr1:9006424-9029979	RpHESP8	*Deltaproteobacteria bacterium*	3e-32	Bacteria
	chr1.0272	Chr1:9011853-9043610	RpHESP7	*Deltaproteobacteria bacterium*	3e-32	Bacteria
	chr1.0273	Chr1:9028231-9063141	hypothetical protein	Acinetobacter pittii	2e-80	Bacteria
	chr1.0274	Chr1:9042744-9049183	RpHESP6	*Deltaproteobacteria bacterium*	2e-32	Bacteria
	chr1.0275	Chr1:9061427-9070020	RpHESP5	*Deltaproteobacteria bacterium*	5e-32	Bacteria
	chr1.0276	Chr1:9093344-9097749	RpHESP4	*Omnitrophica bacterium*	1e-23	Bacteria
	chr1.0277	Chr1:9113101-9118689	RpHESP3	*Omnitrophica bacterium*	3e-14	Bacteria
	chr1.0278	Chr1:9129718-9150751	RpHESP2	*Nocardia albiluteola*	2e-13	Bacteria
	chr1.0279	Chr1:9156360-9157201	uncharacterized protein	*Nilaparvata lugens*	2e-81	Insect
	chr1.0280	Chr1:9182646-9200396	aminopeptidase N	*Halyomorpha halys*	5e-88	Insect
	chr1.0281	Chr1:9208533-9215745	SH3 domain-containing protein Dlish	*Nezara viridula*	0.0	Insect
	chr1.0282	Chr1:9358097-9358759	unknown secreted protein	*Halyomorpha halys*	2e-09	Insect
	chr1.0283	Chr1:9427757-9477787	inorganic phosphate cotransporter	*Nezara viridula*	1e-120	Insect
Riptortus	chr1.0512	Chr1:23494176-23495716	*Riptortus pedestris*-specific protein	-	-	-
pedestris	chr1.0513	Chr1:23502265-23507568	*Riptortus pedestris*-specific protein	-	-	-
**Region2**	chr1.0514	Chr1:23515021-23526711	*Riptortus pedestris*-specific protein	-	-	-
	chr1.0515	Chr1:23530572-23548403	*Riptortus pedestris*-specific protein	-	-	-
	chr1.0516	Chr1:23550702-23555606	*Riptortus pedestris*-specific protein	-	-	-

	chr1.0517	Chr1:23563626-23571711	RpHESP1	*Omnitrophica bacterium*	2e-103	Bacteria
	chr1.0518	Chr1:23579502-23610767	bifunctional purine biosynthesis	*Halyomorpha halys*	0.0	Insect
	chr1.0519	Chr1:23618623-23621122	ATP-dependent (S)-NAD(P)H-hydrate dehydratase	*Halyomorpha halys*	5e-164	Insect
	chr1.0520	Chr1:23621176-23646432	ATP-dependent Clp protease ATP-binding subunit	*Halyomorpha halys*	0.0	Insect
	chr1.0521	Chr1:23654363-23668309	integrator complex subunit 3	*Halyomorpha halys*	0.0	Insect
	chr1.0522	Chr1:23674375-23677715	ribosome biogenesis protein	*Halyomorpha halys*	0.0	Insect
***Oncopeltus fasciatus* Region1**	OFAS000231	Scaffold5:1527122-1544531	cilia- and flagella-associated protein	*Halyomorpha halys*	8e-67	Insect
	OFAS000232	Scaffold5:1554488-1584554	cilia- and flagella-associated protein	*Halyomorpha halys*	4e-55	Insect
	OFAS000233	Scaffold5:1565224-1563695	craniofacial development protein	*Diaphorina citri*	0.0	Insect
	OFAS000234	Scaffold5:1607412-1601077	Chromatin modification protein	*Halyomorpha halys*	0.0	Insect
	OFAS000235	Scaffold5:1615123-1619126	60S ribosomal protein L6	*Halyomorpha halys*	2e-135	Insect
	OFAS000236	Scaffold5:1697851-1702055	OnHESP1	*Omnitrophota bacterium*	2e-21	Bacteria
	OFAS000237	Scaffold5:1712146-1714358	OnHESP2	*Omnitrophota bacterium*	2e-22	Bacteria
***Oncopeltus fasciatus* Region2**	OFAS005040	Scaffold45:127813-126617	ankyrin-1-like	*Wolbachia endosymbiont*	*2e-70*	Bacteria
	OFAS005041	Scaffold45:148698-157666	charged multivesicular body protein	*Halyomorpha halys*	1e-140	Insect
	OFAS005042	Scaffold45:167739-172630	*Oncopeltus fasciatus*-specific protein	-	-	-
	OFAS005043	Scaffold45:185371-180113	*Oncopeltus fasciatus*-specific protein	-	-	-
	OFAS005044	Scaffold45:194910-194800	*Oncopeltus fasciatus*-specific protein	-	-	-
	OFAS005045	Scaffold45:210959-217408	OnHESP3	*Omnitrophota bacterium*	4e-96	Bacteria
	OFAS005046	Scaffold45:246968-252711	*Oncopeltus fasciatus*-specific protein	-	-	-
	OFAS005047	Scaffold45:263991-271095	Collagen alpha-1(XXI) chain	*Papilio xuthus*	8e-23	Insect
	OFAS005048	Scaffold45:272936-273058	*Oncopeltus fasciatus*-specific protein	-	-	-
	OFAS005049	Scaffold45:282097-279948	*Oncopeltus fasciatus*-specific protein	-	-	-
	OFAS005050	Scaffold45:289257-293113	*Oncopeltus fasciatus*-specific protein	-	-	-
***Oncopeltus fasciatus* Region3**	OFAS008428	Scaffold607:69348-88116	partitioning defective 3 homolog	*Nezara viridula*	2e-68	Insect
	OFAS008429	Scaffold607:211962-211117	fork head domain transcription factor	*Halyomorpha halys*	4e-161	Insect
	OFAS008430	Scaffold607:290937-289012	protein sel-1 homolog	*Halyomorpha halys*	5e-168	Insect

	OFAS008431	Scaffold607:321950-322292	unnamed protein product	*Nezara viridula*	5e-05	Insect
	OFAS008432	Scaffold607:366108-372156	OnHESP4	*Omnitrophota bacterium*	6e-46	Bacteria

[1] Gene loci was displayed based on the reported genome of *Riptortus pedestris* and *Oncopeltus fasciatus*. Five genes adjacent to HESPs were displayed.

[2] The best matched species was identified using BLASTp search against NCBI nr database. The top one matched species, excluding Eutrichophora species, was displayed for each gene. Genes that specifically identified in *R. pedestris and O. fasciatus* were labeled as "-"

Supplementary Table 3 Identification of HESP homologues in insect species

Superfamily	Family	Species	Tissue	BioProject Accession	Run Accession	Total Base (Gb)	Number of HESP-associated contigs [1]
Coreoidea	Alydidae	*Riptortus pedestris*	Whole insect	PRJNA671796	SRR13306922	9.4	5
		Leptocorisa acuta	Whole insect	PRJNA629998	SRR11669708	11.1	8
		Alydus pilosus	Whole insect	PRJNA272214	SRR1821895	4.5	12
	Coreidae	*Cletus punctiger*	Whole insect	PRJNA629998	SRR11669704	12.3	2
		Hygia opaca	Salivary gland	PRJNA636678	SRR11928511	10.7	10
		Anoplocnemis dallasi	Salivary gland	PRJNA636678	SRR11928512	11.8	nf
		Homoeocerus walkeri	Head and thorax	PRJNA352589	SRR5040258	4.9	4
		Anasa tristis	Whole insect	PRJNA295690	SRR2496604	4.5	16
	Rhopalidae	*Jadera haematoloma*	Whole insect	PRJNA845792	SRR19540506	6.8	6
		Boisea trivittata	Whole insect	PRJNA272221	SRR1821901	5.3	4
Lygaeoidea	Berytidae	*Yemma signatus*	Antennae	PRJNA317710	SRR3348966	9.4	5
		Metatropis rufescens	Whole insect	PRJNA272197	SRR2051503	2.5	2
		Jalysus sp. AD-2014	Whole insect	PRJNA272253	SRR1821926	5.3	9
	Blissidae	*Ischnodemus falicus*	Whole insect	PRJNA272252	SRR1821925	5.8	13
	Colobathristidae	*Phaenacantha australiae*	Whole insect	PRJNA295735	SRR2496649	5.6	6
	Geocoridae	*Geocoris* sp.	Whole insect	PRJNA272244	SRR1821921	4.6	3
	Lygaeidae	*Oncopeltus fasciatus*	Head	PRJNA430099	SRR7404778	1.4	4
		Lygaeus turcicus	Whole insect	PRJNA272260	SRR1821935	4.9	5
		Arocatus melanocephalus	Whole insect	PRJNA272165	SRR2051471	2.2	7
	Malcidae	*Chauliops fallax*	Whole insect	PRJNA272229	SRR1821938	5	1
	Oxycarenidae	*Oxycarenus laetus*	Whole insect	PRJNA688688	SRR13328464	4.7	1
	Pachygronthidae	*Pachygrontha* sp. KJ-2015	Whole insect	PRJNA295733	SRR2496647	5	13
	Rhyparochromidae	*Gyndes* sp.	Head and thorax	PRJNA352589	SRR5137185	5	3
		Ozophora sp. KJ-2015	Whole insect	PRJNA295747	SRR2496661	5.3	9
Pyrrhocoroidea	Largidae	*Macrocheraia grandis*	Head and thorax	PRJNA352589	SRR5040251	4.8	5
		Largus californicus	Whole insect	PRJNA272803	SRR1821933	6.1	4
	Pyrrhocoridae	*Pyrrhocoris apterus*	Salivary gland	PRJNA389968	SRR5667111	4.5	6
		Dysdercus cingulatus	Head and thorax	PRJNA352589	SRR5040256	4.5	6
Aradoidea	Aradidae 、	*Neuroctenus hainanensis*	Head and thorax	PRJNA352589	SRR5137193	4.7	nf
		Aradus betulae	Whole insect	PRJNA272164	SRR2051470	3.4	nf
		Mezira granulata	Whole insect	PRJNA272266	SRR1821939	5.3	nf
Pentatomoidea	Acanthosomatidae	*Acanthosoma*	Adult	PRJNA183205	SRR921561	2.4	nf

		haemorrhoidale					
	Cydnidae	*Sehirus cincutus*	Whole insect	PRJNA272292	SRR1821969	5.1	nf
	Dinidoridae	*Coridius chinensis*	Whole insect	PRJNA648717	SRR12675197	7.3	nf
	Pentatomidae	*Halyomorpha halys*	No attributes	PRJNA248431	SRR1301985	17.2	nf
	Scutelleridae	*Poecilocoris lewisi*	Whole insect	PRJNA608232	SRR11149667	6.8	nf
	Plataspidae	*Megacopta cribraria*	Whole insect	PRJNA490046	SRR7848655	8.7	nf
	Tessaratomidae	*Tessaratoma papillosa*	Missing	PRJNA597412	SRR10767211	6	nf
	Thyreocoridae	*Corimelaena lateralis*	Whole insect	PRJNA272234	SRR1821910	5.6	nf
	Pentatomidae	*Nezara viridula*	Whole insect	PRJNA838574	SRR19221694	12.6	nf
Cimicoidea	Cimicidae	*Cimex lectularius*	Missing	PRJNA264998	SRR14140300	4.2	nf
	Miridae	*Lygus lineolaris*	salivary gland	PRJNA695862	SRR13587032	1.7	nf
	Anthocoridae	*Orius niger*	Whole insect	PRJNA629998	SRR11781474	11.2	nf
	Nabidae	*Nabis subcoleoptratus*	Whole insect	PRJNA272267	SRR1821941	4.7	nf
Reduvioidea	Reduviidae	*Rhodnius prolixus*	brain	PRJNA853796	SRR19889543	4.9	nf
	Pachynomidae	*Aphelonotus fraterculus*	Whole insect	PRJNA272218	SRR1821898	5.4	nf
	Phymatidae	*Phymata pennsylvanica*	Whole insect	PRJNA272280	SRR1821953	5.9	nf
Gerroidea	Gerridae	*Gerris buenoi*	Missing	PRJEB28079	ERR2860623	8.9	nf
	Mesoveliidae	*Mesovelia mulsanti*	Missing	PRJEB28079	ERR2860622	8.5	nf
Membracoidea	Cicadellidae	*Homalodisca vitripennis*	Whole insect	PRJNA275756	SRR1865089	3.4	nf
	Membracidae	*Entylia carinata*	Missing	PRJNA415461	SRR9942946	4.7	nf
Fulgoroidea	Delphacidae	*Nilaparvata lugens*	Whole insect	PRJNA883205	SRR21720405	7.0	nf
	Cixiidae	*Tachycixius pilosus*	Whole insect	PRJNA272206	SRR2051512	2.4	nf
Coccoidea	Coccidae	*Ericerus pela*	Whole insect	PRJNA551363	SRR9617901	5.8	nf
	Kerriidae	*kerria chinensis*	Whole insect	PRJNA489372	SRR7786294	6.1	nf
Aphidoidea	Aphididae	*Acyrthosiphon pisum*	Whole insect	PRJNA892899	SRR22261921	6.1	nf
	Anoeciidae	*Anoecia corni*	Whole insect	PRJNA295702	SRR2496616	4.4	nf
Psylloidea	Liviidae	*Diaphorina citri*	Whole insect	PRJNA857870	SRR20083769	6.5	nf
	Psyllidae	*Cacopsylla elanoneura*	Missing	PRJEB33150	ERR3386232	9.7	nf
Aleyrodoidea	Aleyrodinae	*Bemisia tabaci*	Whole insect	PRJNA1045855	SRR26978395	4.7	nf

[1] The number of HESP-associated contigs in each species was listed. nf, not find.

Supplementary Table 4 Peptides of RpHESPs identified in salivary sheath using LC-MS/MS

Gene	Unique peptide		Peptides
	Number	Coverage	
RpHESP1	45	47.8%	AGFTTDNVLTLK; AGFTTDNVLTLKK; ARYEPASLISLAK; ASDIIAIHEAGIPAYDIITVSR; DIITLVTSGFK; DILQLSQAGISANDVISFTK; DLLTLTEVGVAPNDIISLTK; EGFVANDISFLR; EIIHLTK; ELLSLVSTGLLAK; EVLSLLHLGLTASDVLSLR; EVLSLLHLGLTASDVLSLRK; FKVNEIVSLR; GGVLGVGSASAVGVGGANAAAASAHSHTK; GKEVLSLLHLGLTASDVLSLR; ITSLINVGVGVR; KSGFAASDLLGLSK; KSGVSTSDIIQLSK; KVGFQSSEITTLTK; NGLVVDEILAISK; PSDLISLVK; QAAHFGLY; SDLVTLVK; SESSQSESSSSSSSSSASSFVSHQDAQLQFLHK; SGVVTSDLLVLVR; SGYSAYDIVSLQSNSGR; SGYSVSDVLTLSK; SLVSIGLK; SPDLFALNR; SSSNSSSNSSSSSSNSAQALGAAALHR; SVGFEVPDLLSLR; SVGFEVPDLLSLRK; TGFSSSEIISLTK; TGLLTDDIITLK; TGLLTDDIITLKK; TGLSTANISSLIK; TSEIVSLTR; TVGLDAEK; VGFNTQQIISLK; VGFQSSEITTLTK; VGLSAPTVISIAR; VNEIVSLR; VSDIISLSQTK; YEPASLISLAK; YSLTPTDIISLSQLGVATK
RpHESP2	12	29.9%	AGISHTSIESLLK; AGISVSQYSTVLQK; AQEIQSLCESGFNTQQLVSLK; EVQQKPAPCGGGEGGCK; GCSSSDLLTLAR; GIATTDIISLAK; HTGFAVPK; IGFKDNDLVSLVSK; KGIATTDIISLAK; LSSSSQGVVTNELTSLLQSGYK; SGISAGTIVK; SGLSVSDVVSVSQCGLPSTK
RpHESP3	9	24.9%	DIISLSNVGFASSHIISLTR; QGYSASQIISSSSQSYQSSESR; SAASSTANAAATAK; SASAQSSASSSSSAAYQGAAGAYAEGAAESAAEISI; SQEIISLTNVGLAAEHIVTLTK; SSSSASASASAAASAESDASSSSQAK; SSSSASSYISSSQQSILQQAGITSSTLSVLAR; VGLSASQLISLTR; VVTLVQEGYSAQDIISFFQR
RpHESP4	16	40.3%	DILSLVK; IVSLATQGLSAQEIISAWSSASK; QAGITSSTLSVLSR; QIISLTNVGLAVPQIVTLTR; SAYAGAANSASAR; SGFSASNILTVLDAGLTTNDLISVVR; SSSISSSQQSILQQAGISTSTLSVLAR; TNDIISLTNLGLASSQIVTLR; TNDIISLTNLGLASSQIVTLRK; ASSSQSSSSSSQQTILR; DILSLAQVGLK; DILSLAQVGLKTK; SGISTSDVLSLVDYGLVGR; SLSSKSSSSSSSSSSSQQAILQQAGISSSTLSVLAR; SVGLYGSQVNQGLSSR; VGVPASQVVSLVQSGYSAQQIISSWSSSSK
RpHESP5	16	49.6%	ASSSQSASSSSSSSSSSQQAILK; DILSLAQVGLR; DILSLAQVGLRTK; DILSLVK; IVSLATQGLSAQEIISAWSSASK; QAGITSSTLSVLSR; QIISLTNVGLAVPQIVTLTR; SAYAGAANSASAR; SGFSASNILTVLDAGLTTNDLISVVR; SGISTNEVLTLVNNGLVGQDILSLAQVGLK; SSSISSSQQSILQQAGISTSTLSVLAR; TNDIISLTNLGLASSQIVTLR; TNDIISLTNLGLASSQIVTLRK; CGLTTSNVLSVIDAGLTASDLISVVK; SGFSASNILTVLHSGLSTSDLLSVVR; VGVPASQVVSLAQSGYSAQQIISSWSSSSK
RpHESP6	18	50.8%	ASSSQSASSSSSSSSSSQQAILK; DILSLAQVGLR; DILSLAQVGLRTK; DILSLVK; IVSLATQGLSAQEIISAWSSASK; QAGITSSTLSVLSR; QIISLTNVGLAANQIVTLTR; QIISLTNVGLAVPQIVTLTR; SAYAGAANSASAR; SGFSASNILTVLDAGLTTNDLISVVR; SGISTNEVLTLVNNGLVGQDILSLAQVGLK; SSSISSSQQSILQQAGISTSTLSVLAR; TNDIISLTNLGLASSQIVTLR; TNDIISLTNLGLASSQIVTLRK; AISGYASQVSQGLSSR; CGLTTSNVLSVIDAGLTASDLISVVK; SGFSASNILTVLHSGLSTSDLLSVVR; VGVPASQVVSLAQSGYSAQQIISSWSSSSK
RpHESP7	18	47.9%	ASSSQSASSSSSSSSSSQQAILK; DILSLAQVGLR; DILSLAQVGLRTK; DILSLVK; IVSLATQGLSAQEIISAWSSASK; QAGITSSTLSVLSR; QIISLTNVGLAVPQIVTLTR; SAYAGAANSASAR; SGFSASNILTVLDAGLTTNDLISVVR; SSSISSSQQSILQQAGISTSTLSVLAR; TNDIISLTNLGLASSQIVTLR; TNDIISLTNLGLASSQIVTLRK; AISGYASQVNQGLSSR; CGLTTSNVLSVIDAGLTASDLISVVK; DILSLAQVGLK; QIISLTDVGLAANQIVTLTR; SGFSASNILTVLHSGLSTSDLLSVVR; VGVPASQVVSLAQSGYSAQQIISSWSSSSK

RpHESP8	16	44.1%	ASSSQSASSSSSSSSSSQQAILK; DILSLAQVGLR; DILSLAQVGLRTK; DILSLVK; IVSLATQGLSAQEIISAWSSASK; QAGITSSTLSVLSR; QIISLTNVGLAANQIVTLTR; QIISLTNVGLAVPQIVTLTR; SAYAGAANSASAR; SGFSASNILTVLDAGLTTNDLISVVR; SGISTNEVLTLVNNGLVGQDILSLAQVGLK; SGISTSDVLTLVDNGLVGR; SSSISSSQQSILQQAGISTSTLSVLAR; SSSSQSSSSSSSSSSSSQQAILQQAGISSSTLSVLAR; TNDIISLTNLGLASSQIVTLR; TNDIISLTNLGLASSQIVTLRK
RpHESP9	9	17.1%	AVSSYPLRPYNYGVLPR; DLLALVR; IPLTVSSSSSQSSSFSGHASSPGSQTVKK; LANAGVEPK; RSSVAPFSALPR; SSGTLPSGASVTTILAQSK; SSVAPFSALPR; TITHSNSVSGR; TLSHSSSSLSGSGDDDVVK

Supplementary Table 5 Peptides of PaHESPs identified in infested clover seed using LC-MS/MS

Gene	Unique peptide		Peptides[2]
	Number[1]	Coverage	
PaHESP1	62	47.4%	AADLVSLAR; AEDLLSLR; AGFEASDVVFLK; AGFEASDVVFLKR; AGFSNSQVSLLVQSGYTK; AGGLSAYGAYGAAQGAHQVAR; AGHTAYDICSMNR; AGLVSNDIIFLSR; AGVAGLSNVGVSQGVLGYGSR; ANDIISLTK; DAVALTR; DCLSLTSVGLK; DIFTLRK; DIVSLTK; DLISFTK; DLLSLASSGLK; DLLTITK; DVISLTSVGIAPR; EAGLTSNDIISVSR; GLVATDIITLSK; GYAVTDIVSLSQTK; GYGANDLLTLRR; IGLDAPQIVAITK; KGLVATDIITLSK; KGYAVTDIVSLSQTK; KSNFIAQDIISLCSTGFR; LSSQEIVHLAR; NNMYAGAAAAK; QIIALTK; RFGLSAK; SAGLAANDIQSLIK; SESSSSHSSSHSSHNLAAGYHGAR; SGFASSDLIK; SGFVAPQIISLSK; SGFVASDLITLTK; SGFVASDLLSLTK; SGLFANDIISLSR; SGLIVNDIVSLGR; SGLTASYVISMSR; SIVSLTGVGLK; SKGYGANDLLTLR; SKGYGANDLLTLRR; SLLTVVQTGYK; SSELITVLR; SSSSSQSSSSHSSSSYTSQQNVK; STDILTLVR; STDLLSLVK; STEILSLSNSGLSTEQIITLSK; STEIVTAR; STELLTLHK; SVGLDSER; TASEILSLLK; TEELISTIK; TGFTSSNLLTLATSGLK; TGFVANDLLTLTR; TLVNAGINTK; TTDITFLSNSGLAPK; TTDLVSLVR; TTDLVTLVR; VGFSATNIVSLK; VGFSATNIVSLKR; VGFTATDIISIK
PaHESP2	14	55.2%	AEDIISLSR; GCAAGDLISLSR; NGFRAEDIISLSR; QLISLTSVGYAPVDIVTISR; SGLSVNDVIK; SKSQSNSQSNSLSAVLSK; SQSNSQSNSLSAVLSK; SSSTQSEESAASSSSSSAMK; TDDVVALAQVGLVAR; TTSGVITEELLSLTR; VGFNAEQVLPLCNAGFSSK; VSQCGLEPAR; VVSLVQSGHSASQIVSYSYSSK; VVSLVQSGHSASQIVSYSYSSKSSSSK
PaHESP3	47	62.3%	AGFDALDIVR; AGIFPNELSYLSGHGIQPSDALTLVNLGLPAK; AGLSPSQLSVLANSGVAPTQVLDLVR; ATSQENAPQTQSPVSDQPSSPK; DIITLAR; DILSLAK; DILSLANIGLR; DIVSVAR; DLVTVCGSGLSAR; EIVSIGR; HAGFTKPQISTLSR; IEVIGLLEHGLTSR; IGFAAPQIVSLYNVGYHVPELMTLAR; LGLPSSQVVSLVR; LGYSVPQIMSFVSPNLSLVQPTIHHVTHYVPNTIPIQR; LVSPVTQSFSSR; NESPGSSEPNSSDSSPSSPEPNDVLPAR; NVHVSIPSQVAHYQHVVPTIHQNVR; QVAETLPK; RPQVSTPK; RVVSLVR; SENVQVNNPSER; SFNVASSPDFASSFQMVTALQK; SGYSANDIAK; SGYTVPEILSLTESKPNVR; SNKPTSQGNAPQTESPVSGQPGSPK; SNRPAGQENVSQTESTVTEQPGSPK; SNRPTGQENVPQTESSVSR; SPVEEQPSSSTSSPR; SQSSQVLSFLR; SRPQSESLVSSQISSPPLMPSKPK; SSIQVGGR; SSTSTSENVQVNRPSEVK; SSTSVSEVSTAEASVSPQPGTSHESPSRPR; STPVVEAPVSPQSGASHESPSRPR; STSQVEATSSPKPEVSHESPSRPR; TNDILSLTK; TSTANSENVQVNSPSEVR; TSTATSESVQVNSPSEVNNTPR; TTEISSLADIGLAAPEIVSLTK; VEATSSPKPEVSHESPSRPR; VGFSAPQLFTISK; VGLCGLPAK; VGLCGLPAKR; VPEPINSR; VSEASTVDAPVSPQPVASHESPSRPR; YFNQISHTSSVSSSSGIR
PaHESP4	33(10)	49.7%	AAAVGKQTAAVAK; AGASVAAPAVR; AGLSAQEIVTVGR; AVAGAAVGR; CGLAANK; CGLAANKVVSLVNNGYSASDIVSVAR; CGLAANKVVSLVNNGYSASDIVSVARR; KAAAVGKQTAAVAK; QASSQSSSSQSSQSSSSSSESVALSR; RDILSVAR; SGAASQLAANAAR; SGAASQLAANAARK; SSASQSSQSSSAQSSEASSASASEAASVGVSGASAVR; SSKSVSSSSSSSSSYEASASSQQSAVLQR; SSSSHSSSSEASSSESLASDAAYGASAAGYNAAAYGY; SVSSSSSSSSSYEASASSQQSAVLQR; TGFSATDLVSVCSAGWSR; TGFSATDLVSVCSAGWSRR; TNQIVSLTSIGLAAPQIVR; VGLAVPQIVSLCQVGFAAPQLLTLTR; VGLRTNQIVSLTSIGLAAPQIVR; VVSLVNNGYSASDIVSVAR; VVSLVNNGYSASDIVSVARR; AGFTSSQVSLLANLGLPTTEVVSLVEYGLPSNDIISLAR; AGISTSQLSVLAR; DILSVAR; LGLPAEQVVSLVQSGYSAADVQEYSYQSR; SGISRTDVLSLVNVGLVGNDILSLAR; TDVLSLVNVGLVGNDILSLAR; TNEIISLTR; VGFSAADLIGISR; VGLRTNEIISLTR; VGLTSSDLLSLTR
PaHESP5	--	--	--

PaHESP6	26(10)	49.0%	AGLTAQEIVTVGR; AKAVTGAAVGR; AVTGAAVGR; IGYSATDLVSVCSAGWSSR; QAAVSQQAASSR; SASSQSASSESSQSSSSSSSSSSESVALSR; SSASQSSQSSSAESSEASSASSEAASVGVSGASAVSAGAAVAVPAVR; SSKSSSSQSSHSSSSSSSYEASASSQQSAVLQR; SSSSHSSSSEASSSESLASDAAYGASAAGYGAAAWY; SSSSQSSHSSSSSSSYEASASSQQSAVLQR; SSSSSSKSSSSHSSSSEASSSESLASDAAYGASAAGYGAAAWY; TNQIISLTDIGLAAPQIVSLTR; VGLAAPQIVSLCQVGFAAPQLLTLTR; VGLRTNQIISLTDIGLAAPQIVSLTR; VVSLVNSGASVSDIVSVAR; VVSLVNSGASVSDIVSVARR; AGFTSSQVSLLANLGLPTTEVVSLVEYGLPSNDIISLAR; AGISTSQLSVLAR; DILSVAR; LGLPAEQVVSLVQSGYSAADVQEYSYQSR; SGISRTDVLSLVNVGLVGNDILSLAR; TDVLSLVNVGLVGNDILSLAR; TNEIISLTR; VGFSAADLIGISR; VGLRTNEIISLTR; VGLTSSDLLSLTR

[1] The number in the brackets indicates unique peptides belonging to more than one protein.

2 Unique peptides belonging to more than one protein are labelled in red.

Supplementary Table 6 Sequences used in phylogenetic analysis

Gene	Species	ID
RpHESP1	*Riptortus pedestris*	OQ126870
RpHESP2	*Riptortus pedestris*	OQ126869
RpHESP3	*Riptortus pedestris*	OQ126868
RpHESP4	*Riptortus pedestris*	OQ126867
RpHESP5	*Riptortus pedestris*	OQ126866
RpHESP6	*Riptortus pedestris*	OQ126865
RpHESP7	*Riptortus pedestris*	OQ126864
RpHESP8	*Riptortus pedestris*	OQ126863
RpHESP9	*Riptortus pedestris*	OQ126862
PaHESP1	*Pyrrhocoris apterus*	OQ126871
PaHESP2	*Pyrrhocoris apterus*	OQ126872
PaHESP3	*Pyrrhocoris apterus*	OQ126873
PaHESP4	*Pyrrhocoris apterus*	OQ126874
PaHESP5	*Pyrrhocoris apterus*	OQ126875
PaHESP6	*Pyrrhocoris apterus*	OQ126876
OfHESP1	*Oncopeltus fasciatus*	OFAS005045
OfHESP2	*Oncopeltus fasciatus*	OFAS008432
OfHESP3	*Oncopeltus fasciatus*	OFAS000236
OfHESP4	*Oncopeltus fasciatus*	OFAS000237
hypothetical protein	*Actinomycetales bacterium*	TEX51455
hypothetical protein	*Deltaproteobacteria bacterium*	RLB68275
hypothetical protein	*Omnitrophica bacterium*	MBI4549786
hypothetical protein	*Thorarchaeota archaeon*	RLI49244
hypothetical protein	*Candidate* division CPR1 bacterium	KKS44764
hypothetical protein	*Omnitrophica WOR_2 bacterium*	OGX24733
hypothetical protein	*Pirellulales bacterium*	MCD4727973
hypothetical protein	*Coatesbacteria bacterium*	RLC39126
hypothetical protein	*Shewanella gelidimarina*	WP_248989019
hypothetical protein	*Ferrimonas balearica*	WP_222524125
hypothetical protein	*Marinobacter nauticus*	WP_222204063
hypothetical protein	*Shewanella schlegeliana*	WP_202721438
hypothetical protein	*Elusimicrobia bacterium*	NLE65710
hypothetical protein	*Pelotomaculum* sp.	OPX89066

Supplementary Table 7 Primers used in this study

Gene	Forward primer (5'-3')	Reverse primer (5'-3')
**Primers used in dsRNA synthesis**		
RpHESP1	TAATACGACTCACTATAGGGATGAGGCTCTGTCTGGTCATAA	TAATACGACTCACTATAGGGGATTTCCTTGCAAGATAATC
RpHESP2	TAATACGACTCACTATAGGGATGTGGAGGTGGAGAAGGTGG	TAATACGACTCACTATAGGGTCCTCCATTGGATGCAGGTCTA
RpHESP3	TAATACGACTCACTATAGGGATGACCATGCGTGCATCAGTGA	TAATACGACTCACTATAGGGAGGTGATGCCTGCTTGCTGG
RpHESP4-8	TAATACGACTCACTATAGGGGATCTCAGGTGAGTCAGGGA	TAATACGACTCACTATAGGGGAGGACGTCAGAAGTGGAGA
RpHESP9	TAATACGACTCACTATAGGGATGACTGGAATTCTGCCGCTC	TAATACGACTCACTATAGGGGACAACGTTTTGGACAGTGCCG
PaHESP1	TAATACGACTCACTATAGGGGGTTCTTCAGCTGCCATCAAC	TAATACGACTCACTATAGGGGACAGCAGATCTGTTGACTTG
PaHESP2	TAATACGACTCACTATAGGGGTTAACACAGGTTGGCATCAC	TAATACGACTCACTATAGGGGAACTCTTTTTGGACGAACTGG
PaHESP3	TAATACGACTCACTATAGGGTTTGGGACTGAGTTCCGTTTC	TAATACGACTCACTATAGGGGGCTGTGACACTTGTGGAACT
PaHESP4	TAATACGACTCACTATAGGGGAAAACCACTCTGGTCTTAGC	TAATACGACTCACTATAGGGTGCTCCTACAGAACCAACTCC
PaHESP5	TAATACGACTCACTATAGGGAGTATTCAGCCGACGCGAGAT	TAATACGACTCACTATAGGGCAGCAGGAACTGGAAATGAAG
PaHESP6	TAATACGACTCACTATAGGGATGCGTACCTCCGTACTAGCT	TAATACGACTCACTATAGGGCAGATCTCACTGCTCCAACAG
GFP	TAATACGACTCACTATAGGGAGAATGAGTAAAGGAGAAGAACTTTTC	TAATACGACTCACTATAGGGAGATTTGTATAGTTCATCCATGCCATGT
**Primers used in qRT-PCR**		
RpHESP1	CGTCGGAAATAACAACTCTG	TGGAATACCTGCTTCGTGGA
RpHESP2	CTGACTCTGGCAAGAAGTGGCA	TATGCTTGACTGGAAGCAGCAG
RpHESP3	TTCACTCACAAATGTCGGTCTA	TTACTACTCGCTGTGCTGGA
RpHESP4-8	TCCCAATCATCATCTTCATC	CCTACGAGTCCGTTATCTAC
RpHESP9	TCTGGTGGAGACGGTCCTAA	CGAATCCGATGGTTCATTTT
RpActin	CTCCTGAGTCAAGCACAATA	GCATCACACCTTCTACAATG
RpGAPDH	CTGTTGTAGACCTCACTGTT	ATATCCGCACTCATTGTCAT
PaHESP1	CTACAACAGAGGTCATATCAG	CTACGAAGCCAGACTTAATAG
PaHESP2	CAGTAAGAAGTCAGGAGGTTG	TTAACTGGCTGTACTGTTCTG
PaHESP3	TAAGTCACCAACCAGAGTATC	CTATTAGAGCGAGGTCTTGAA
PaHESP4	TGATGTTGGTGGCTATGC	GGACAGACTCTTCCTTGC
PaHESP5	AATCAGAGCGAAGAAGGTTAC	GACGATATACCAGAACTGTGA
PaHESP6	GCTCTCAATCTGAAGAATCTG	GATGATGACGAGGAAGATGAG
PaActin	CGTGACTTGACCGACTAC	GTGGATACCGCAGGATTC
PaGAPDH	GAAGCAGTGTTACCGATGA	TTGTGGCGAGATGGAAGA
