# Supplementary material for: Salivary proteins potentially derived from horizontal gene transfer are critical for salivary sheath formation and other feeding processes
Source: Commun Biol. 2024 Mar 2;7:257. doi: 10.1038/s42003-024-05961-9 (PMC10908841; doi:10.1038/s42003-024-05961-9)
Supplement: Supplementary file 5 — Reporting Summary [file 42003_2024_5961_MOESM5_ESM.pdf]

nature portfolio

Corresponding author(s): Hai-Jian Huang
Last updated by author(s): 2023-12-7

Reporting Summary

Nature Portfolio wishes to improve the reproducibility of the work that we publish. This form provides structure for consistency and transparency in reporting. For further information on Nature Portfolio policies, see our Editorial Policies and the Editorial Policy Checklist.

Statistics

For all statistical analyses, confirm that the following items are present in the figure legend, table legend, main text, or Methods section.

n/a	Confirmed	
☐	☒	The exact sample size (*n*) for each experimental group/condition, given as a discrete number and unit of measurement
☐	☒	A statement on whether measurements were taken from distinct samples or whether the same sample was measured repeatedly
☐	☒	The statistical test(s) used AND whether they are one- or two-sided<br>*Only common tests should be described solely by name; describe more complex techniques in the Methods section.*
☒	☐	A description of all covariates tested
☒	☐	A description of any assumptions or corrections, such as tests of normality and adjustment for multiple comparisons
☐	☒	A full description of the statistical parameters including central tendency (e.g. means) or other basic estimates (e.g. regression coefficient) AND variation (e.g. standard deviation) or associated estimates of uncertainty (e.g. confidence intervals)
☐	☒	For null hypothesis testing, the test statistic (e.g. *F*, *t*, *r*) with confidence intervals, effect sizes, degrees of freedom and *P* value noted<br>*Give P values as exact values whenever suitable.*
☒	☐	For Bayesian analysis, information on the choice of priors and Markov chain Monte Carlo settings
☒	☐	For hierarchical and complex designs, identification of the appropriate level for tests and full reporting of outcomes
☒	☐	Estimates of effect sizes (e.g. Cohen's *d*, Pearson's *r*), indicating how they were calculated

Our web collection on statistics for biologists contains articles on many of the points above.

Software and code

Policy information about availability of computer code

Data collection	Roche Light Cycler® 480 Real-Time PCR System for qPCR; SEM TM4000 II plus for scanning electron microscopy; Thermo Fisher Q Exactive HF-X mass spectrometer for proteomic data collection.
Data analysis	The softwares used in this study were commonly used. In detail, MaxQuant 1.6.5.0 was used to analysis MS data. SPAdes (v 3.13.0) was used to assemble the transcriptome. The SignalP 5.0 Server (https://services.healthtech.dtu.dk/service.php?SignalP-5.0) was employed to predict the presence of signal peptides and cleavage sites. Splign Server (https://www.ncbi.nlm.nih.gov/sutils/splign/splign.cgi) was utilized to predict genomic structure of ESSP genes. TransDecoder v5.5.0 was used for protein predication. OrthoMCL v2.0.9 was used for orthologous analysis. MegAlign v7.1.0 was adopted to estimate the amino acid sequence similarity. MAFFT was used to perform sequence alignment. ModelTest-NG was employed to determine the best model (PROTGAMMAJTT). Bayesian MCMCTree v4.9i was used to perform the divergence time analysis. ImageJ software v1.53e was used to measure salivary sheath length.

For manuscripts utilizing custom algorithms or software that are central to the research but not yet described in published literature, software must be made available to editors and reviewers. We strongly encourage code deposition in a community repository (e.g. GitHub). See the Nature Portfolio guidelines for submitting code & software for further information.

Data

Policy information about availability of data

All manuscripts must include a data availability statement. This statement should provide the following information, where applicable:

- Accession codes, unique identifiers, or web links for publicly available datasets
- A description of any restrictions on data availability
- For clinical datasets or third party data, please ensure that the statement adheres to our policy

The mass spectra data generated in this study were submitted to the ProteomeXchange with accession numbers PXD039150 and PXD039269. Sequences of PaESSPs and RpESSPs have been deposited in NCBI GenBank with accession numbers OQ126862-OQ126876.

Research involving human participants, their data, or biological material

Policy information about studies with human participants or human data. See also policy information about sex, gender (identity/presentation), and sexual orientation and race, ethnicity and racism.

Reporting on sex and gender	not applicable
Reporting on race, ethnicity, or other socially relevant groupings	not applicable
Population characteristics	not applicable
Recruitment	not applicable
Ethics oversight	not applicable

Note that full information on the approval of the study protocol must also be provided in the manuscript.

Field-specific reporting

Please select the one below that is the best fit for your research. If you are not sure, read the appropriate sections before making your selection.

☒ Life sciences ☐ Behavioural & social sciences ☐ Ecological, evolutionary & environmental sciences

For a reference copy of the document with all sections, see nature.com/documents/nr-reporting-summary-flat.pdf

Life sciences study design

All studies must disclose on these points even when the disclosure is negative.

Sample size	No statistic method was used to pre-determine the sample size. Sample sizes were chosen based on established protocols and previous publications. Generally three independent biological replicates were done for each experiment. The insect bioassays (survival, feeding time) were referred to Huang et al., 2023 (10.1038/s41467-023-36403-5).
Data exclusions	No data was excluded from the analyses.
Replication	All attempts at replication were successful. Experiments were repeated three times.
Randomization	All samples were allocated randomly into experimental groups.
Blinding	All investigation were blinded to group allocation during data collection and analysis

Reporting for specific materials, systems and methods

We require information from authors about some types of materials, experimental systems and methods used in many studies. Here, indicate whether each material, system or method listed is relevant to your study. If you are not sure if a list item applies to your research, read the appropriate section before selecting a response.

Materials & experimental systems

n/a	Involved in the study
☒	☐ Antibodies
☒	☐ Eukaryotic cell lines
☒	☐ Palaeontology and archaeology
☐	☒ Animals and other organisms
☒	☐ Clinical data
☒	☐ Dual use research of concern
☒	☐ Plants

Methods

n/a	Involved in the study
☒	☐ ChIP-seq
☒	☐ Flow cytometry
☒	☐ MRI-based neuroimaging

Animals and other research organisms

Policy information about studies involving animals; ARRIVE guidelines recommended for reporting animal research, and Sex and Gender in Research

Laboratory animals	Riptortus pedestris was originally collected from a soybean field (33.7° N, 117.0 °E) in Suzhou, China, and reared on soybean seeds for more than 20 generations. Pyrrhocoris apterus was originally collected from Xinjiang, China, and reared on clover seeds.
Wild animals	This study did not involve wild animals.
Reporting on sex	Not appliable
Field-collected samples	This study did not involve samples collected from field.
Ethics oversight	No ethical approval or guidance was required.

Note that full information on the approval of the study protocol must also be provided in the manuscript.
